# Policy Recommendations From Transmission Modeling for the Elimination of Visceral Leishmaniasis in the Indian Subcontinent

**DOI:** 10.1093/cid/ciy007

**Published:** 2018-06-01

**Authors:** Epke A Le Rutte, Lloyd A C Chapman, Luc E Coffeng, José A Ruiz-Postigo, Piero L Olliaro, Emily R Adams, Epco C Hasker, Marleen C Boelaert, T Deirdre Hollingsworth, Graham F Medley, Sake J de Vlas

**Affiliations:** 1 Department of Public Health, Erasmus MC, University Medical Center Rotterdam, The Netherlands; 2 Zeeman Institute, University of Warwick, Coventry, United Kingdom; 3 London School of Hygiene and Tropical Medicine, United Kingdom; 4 Department of Control of Neglected Tropical Diseases; 5 Special Programme for Research and Training in Tropical Diseases, World Health Organization, Geneva, Switzerland; 6 Liverpool School of Tropical Medicine, United Kingdom; 7 Institute of Tropical Medicine, Antwerp, Belgium; 8 Big Data Institute, Li Ka Shing Centre for Health Information and Discovery, University of Oxford

**Keywords:** visceral leishmaniasis, Indian subcontinent, transmission modeling, WHO guidelines, elimination

## Abstract

**Background:**

Visceral leishmaniasis (VL) has been targeted by the World Health Organization (WHO) and 5 countries in the Indian subcontinent for elimination as a public health problem. To achieve this target, the WHO has developed guidelines consisting of 4 phases of different levels of interventions, based on vector control through indoor residual spraying of insecticide (IRS) and active case detection (ACD). Mathematical transmission models of VL are increasingly used for planning and assessing the efficacy of interventions and evaluating the intensity and timescale required to achieve the elimination target.

**Methods:**

This paper draws together the key policy-relevant conclusions from recent transmission modeling of VL, and presents new predictions for VL incidence under the interventions recommended by the WHO using the latest transmission models.

**Results:**

The model predictions suggest that the current WHO guidelines should be sufficient to reach the elimination target in areas that had medium VL endemicities (up to 5 VL cases per 10000 population per year) prior to the start of interventions. However, additional interventions, such as extending the WHO attack phase (intensive IRS and ACD), may be required to bring forward elimination in regions with high precontrol endemicities, depending on the relative infectiousness of different disease stages.

**Conclusions:**

The potential hurdle that asymptomatic and, in particular, post-kala-azar dermal leishmaniasis cases may pose to reaching and sustaining the target needs to be addressed. As VL incidence decreases, the pool of immunologically naive individuals will grow, creating the potential for new outbreaks.

Visceral leishmaniasis (VL), also known as kala-azar, is a neglected tropical disease caused by protozoan *Leishmania* parasites transmitted by female *Phlebotomine* sandflies. Only a small proportion of infected individuals develop clinical symptoms, which include prolonged fever and an enlarged liver and spleen. VL is generally considered fatal if left untreated [1, 2]. After recovery and, more rarely, after asymptomatic infection, individuals can develop post-kala-azar dermal leishmaniasis (PKDL), a skin rash involving macular, papular, or nodular lesions [3]. Individuals with PKDL are thought to contribute to transmission [3–6].

The largest burden of VL has traditionally been in the Indian subcontinent (ISC), where transmission is considered solely anthroponotic. In 2014 and 2015, however, with conflicts in East Africa, the African region reported more cases than the ISC [7]. The decrease in cases in the ISC has been attributed to Nepal, India, and Bangladesh instituting a program in 2005 to eliminate VL as a public health problem by 2017 [7, 8]. In 2014, Bhutan and Thailand joined the commitment and the target was set for 2020 [9]. The elimination target is annual incidence of <1 VL case per 10000 inhabitants for 3 consecutive years at subdistrict/district level (depending on the country) [2]. The program has 4 phases: a precontrol “preparatory” phase; a 5-year attack phase designed to bring the incidence below 1 per 10000 per year by 2017; a consolidation phase where incidence is kept below the target for 3 years; and a maintenance phase to ensure sustainable reductions in incidence beyond 2020 [10]. These phases entail different levels of intervention activities including active case detection (ACD) and vector control through indoor residual spraying of insecticide (IRS) or other effective vector control measures, which are further explained in the World Health Organization (WHO) guidelines [10].

Mathematical transmission models are increasingly used for planning and assessing the efficacy of interventions for VL, although challenges remain due to key biological uncertainties in its transmission dynamics [11–14]. This article draws together the key policy-relevant conclusions from recent transmission modeling of VL, and presents new predictions of the impact of the interventions in each WHO phase on VL incidence. We also explore alternative durations of these phases, to aid prioritization of resources for VL control in the ISC.

## OVERVIEW OF RECENT VL MODELING AND KEY POLICY-RELEVANT OUTCOMES

There are several published models of VL transmission dynamics [11, 12]. Those which are focused on the ISC have been particularly influenced by the work of Stauch et al [15, 16]. More recently, modeling groups from Erasmus MC and Warwick University have been performing transmission modeling and quantitative analyses in this area [17–19], and we use these models in this article. We first describe the models and highlight the main uncertainties in VL transmission dynamics.

### Description of Transmission Models and Key Knowledge Gaps in VL Dynamics

**Figure 1.**
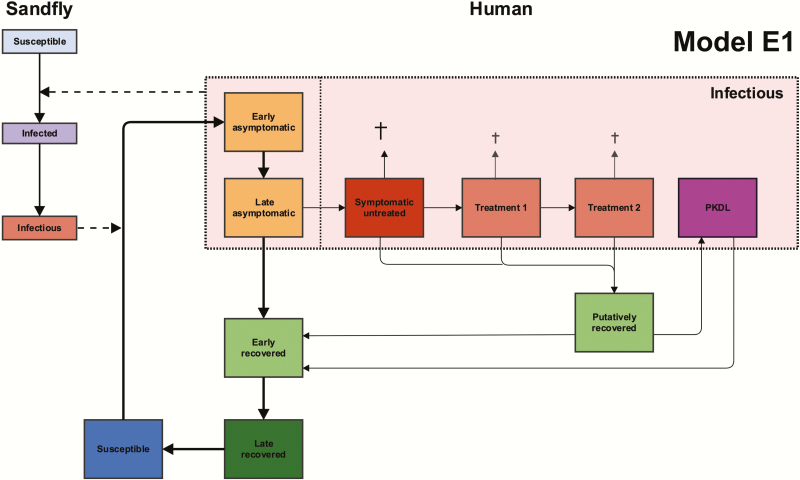
Schematic presentation of the structures of model E1 and the related models E0, W1, and W0. Model W1 is similar to model E1, but has one combined compartment for asymptomatic individuals (yellow), and 1 combined compartment for recovered individuals (green), and no post-kala-azar dermal leishmaniasis (PKDL; purple). For models E1 and W1, asymptomatic individuals (yellow compartments) are the main contributors to transmission. Models E0 and W0 have the same structures as models E1 and W1, respectively, but asymptomatic individuals do not contribute to transmission. All 4 models have different durations of infection stages from fitting to data, which are listed elsewhere [19]. Indoor residual spraying reduces the populations of the sandfly compartments, and active case detection leads to a shorter duration of the symptomatic untreated state (dark red) in all models.

Figure 1 illustrates the basic structure of the Erasmus MC (E0, E1) and Warwick models (W0, W1), with the main differences between models explained in the legend. The models are deterministic, and were parameterized using different data, but have both undergone geographical cross-validation against data on >5000 VL cases from 8 endemic districts in Bihar collected by CARE India [20] (see [19] for full model descriptions and sensitivity analyses).

Attempts to model VL transmission and control have highlighted the importance of certain parameters that are highly variable or remain largely unknown [12, 13, 21]. These parameters can be distinguished according to whether they relate to human aspects of infection, sandfly bionomics, or intervention efficacy. Regarding the human aspects of infection, the key unknown parameters are the duration of asymptomatic infection, the proportion of asymptomatic individuals who develop clinical symptoms, the relative infectiousness of asymptomatic individuals, VL cases, and PKDL cases, and the duration of acquired immunity. Therefore, the models make different assumptions about these aspects of the natural history.

The Erasmus MC models [18, 19] consist of a set of age-structured model variants based on different assumptions about where the main reservoir of infection lies; namely, solely in symptomatic individuals (VL and PKDL; model E0), or mainly in asymptomatic individuals (model E1). Other variants, with the main reservoir of infection in previously immune individuals in whom infection reactivates or PKDL cases, have also been explored [18]. The models were parameterized with age-structured data on approximately 21000 individuals included in the KalaNet bednet trial in India and Nepal [22].

In the original Warwick model (model W1) [19], asymptomatic individuals constitute the main reservoir of infection, and PKDL cases are not included in the transmission dynamics. Certain parameters, such as the durations of asymptomatic infection and immunity, are based on estimates from modeling of the natural history of VL, using annual serological test and skin test results from a detailed epidemiological study in a high-endemicity setting in Bangladesh [17, 23]. Here, we introduce 1 new model variant (model W0), which is comparable to model W1 except that only symptomatic individuals contribute to transmission, as in model E0.

Using these models, and other statistical approaches, attempts have been made to estimate the average durations of asymptomatic infection and immunity. Le Rutte et al [19] reported that asymptomatic infection (defined by polymerase chain reaction positivity without symptoms) lasts approximately 10 months (95% confidence interval [CI], 8–14 months) based on the KalaNet data. Chapman et al [17] concluded that the asymptomatic stage (defined by rK39 enzyme-linked immunosorbent assay positivity and leishmanin skin test negativity without symptoms) lasts approximately 5 months (95% CI, 4–5.5 months), based on the Bangladesh data. The percentage of asymptomatic individuals that develop VL was estimated by Le Rutte et al at approximately 1.5% [19], whereas Chapman et al estimated it at approximately 15% [17]. These estimates may reflect potentially realistic possibilities at both ends of the spectrum [21, 24], and mainly differ because the models had different structures and were fitted to datasets from settings with different endemicities and diagnostics [19, 24].

As there are no definitive data on the duration of immunity to VL after (asymptomatic and symptomatic) infection, in a recent model comparison study [19] it was assumed to last 2 years (by Erasmus MC), based on fitting of the models to the KalaNet and CARE data [18, 19], and 5 years (by Warwick) based on previous modeling of the natural history of VL [17]. However, the potential implications of some individuals developing lifelong immunity to disease remain to be explored.

The relative infectiousness of individuals in different infection states remains under debate [13]. Despite limited data and reliance on strong underlying assumptions, the modelers estimated the infectiousness of asymptomatics from case incidence data as about 2.5% that of symptomatic cases [19]. In models E0 and E1, PKDL cases are assumed to be half as infectious to sandflies as active VL cases, which is considered conservative based on available data [25]. Furthermore, we note that all 3 lesion types contain detectable parasite loads [26], and have been shown to transmit to sandflies in historical and recent xenodiagnosis studies [5, 6]. Ongoing xenodiagnosis studies will provide more direct evidence of the relative infectiousness of asymptomatic individuals and PKDL cases [6, 27]. Detailed longitudinal follow-up studies are required to provide further insight into the progression of asymptomatic individuals to clinical disease and the duration of immunity.

Also, little is known about the bionomics of *Phlebotomus argentipes* sandflies. All 4 models assume the same parameter values for sandfly bionomics [19]. Models E0 and E1 treat exposure to sandflies as age-dependent to explain observed age patterns in seroprevalence and VL incidence [18, 19]. The relationship between sandfly and host densities, prevalences of infected and infectious flies, host biting preferences, time and location of transmission, and *P. argentipes* life expectancy remain largely uncertain [12, 13].

**Policy-Relevant Insights From Recent VL Modeling**

Modeling has shown that reducing time to diagnosis, and subsequent treatment, can lead to a dramatic reduction in incidence of VL cases [19, 28]. The impact of early identification and treatment of VL cases, for example, during fever before the onset of VL-specific symptoms, was explored by Medley et al in a model that assumes that clinical VL cases are significantly more infectious than asymptomatic or preclinical cases [28]. The results highlighted the importance of the timeliness of diagnosis, suggesting that a diagnostic capable of targeting earlier treatment need only be 30% sensitive to have a significant impact. However, such a diagnostic would need to be highly specific to justify VL treatment using current drugs. In this regard, it has been shown that individuals with high initial antibody levels, and those who seroconvert to high antibody levels, are more likely to develop clinical VL than individuals who are seronegative, or who do not seroconvert between tests [17, 24]. However, the specificity of using high-titer seropositivity or seroconversion to identify progressors to VL is low [24].

The impact of IRS was first studied in a model in which asymptomatic individuals are the main drivers of transmission [15]. This suggested that a large reduction in sandfly density via IRS (of around 60%–70%) is needed to achieve elimination. Le Rutte et al later demonstrated that reducing cases below 1 per 10000 per year could be feasible with optimal IRS (63% continuous reduction in sandfly density) in low-endemicity and medium-endemicity settings (≤10 cases per 10000 per year at baseline). In higher-endemicity areas, additional interventions were advised [18]. All models assume constant effectiveness of IRS, which requires that susceptibility to insecticides is monitored and managed, in terms of switching between different insecticides if resistance arises [29].

Recent modeling suggests that if asymptomatic individuals are the main contributors to transmission (models E1 and W1), the continuation of combined IRS and ACD at current levels (60% IRS coverage and 40-day average onset-to-treatment time) should be sufficient to reach the elimination target by 2020 for subdistricts with a precontrol endemicity ≤10 per 10000 per year [19]. However, if transmission is caused solely by symptomatic individuals (model E0), the models suggest the target will be reached years later, due to transmission being maintained by a remaining pool of VL and PKDL cases with long infectious periods. The Erasmus MC and Warwick models gave discrepant results on whether increasing IRS coverage from 60% to 80% or halving the average onset-to-treatment time from 40 to 20 days reduced incidence more rapidly. These discrepancies are largely due to different estimated IRS efficacies and parameterization of asymptomatic infection (relative infectivities and durations), and inclusion of PKDL (in terms of the long-term predictions). All models agreed that a combination of increasing IRS coverage and reducing onset-to-treatment times would lead to the target being reached most quickly, and that these 2 interventions together would be sufficient to achieve the elimination target in all settings.

## IMPACT OF THE DIFFERENT PHASES OF THE WHO GUIDELINES ON VL INCIDENCE

With the 4 transmission models (models E0, E1, W0, and W1) we predict the effect that the different WHO phases have on VL incidence over time for subdistricts with high (10 per 10000 per year), medium (5 per 10000 per year), and low (2 per 10000 per year) precontrol endemicity levels. During the precontrol phase without IRS and ACD, an average “onset-to-treatment” (OT) time of 60 days is assumed [20, 30]. In the attack phase, active case detection is assumed to reduce the OT to 45 days, combined with 100% IRS coverage as mentioned in the guidelines. We interpret this 100% to be comparable to the maximum IRS coverage so far achieved in Bihar, which is equivalent to 67% of all households being sprayed (53% households fully sprayed and 29% partially sprayed [53% + 29% / 2 = 67%]) [31]. The “limited IRS” in the consolidation phase [10] is interpreted here as two-thirds of the IRS coverage in the attack phase, combined with “intensified ACD” leading to a shorter OT of 30 days.

**Figure 2.**
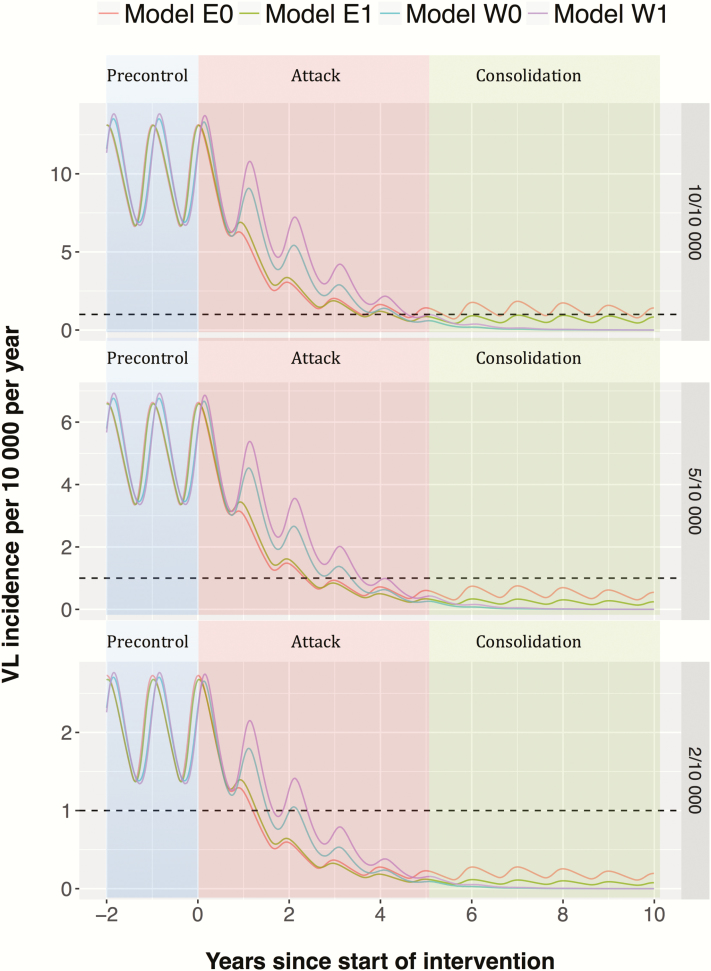
Visceral leishmaniasis (VL) incidence during the World Health Organization precontrol phase (<year 0), attack phase (years 0–5), and consolidation phase (year 5 onward) for 3 different precontrol endemicity levels (2, 5, and 10 cases per 10000 people per year), as predicted from 4 transmission models. Oscillations are due to the seasonal pattern in incidence caused by seasonal variation in the sandfly population.

**Figure 3.**
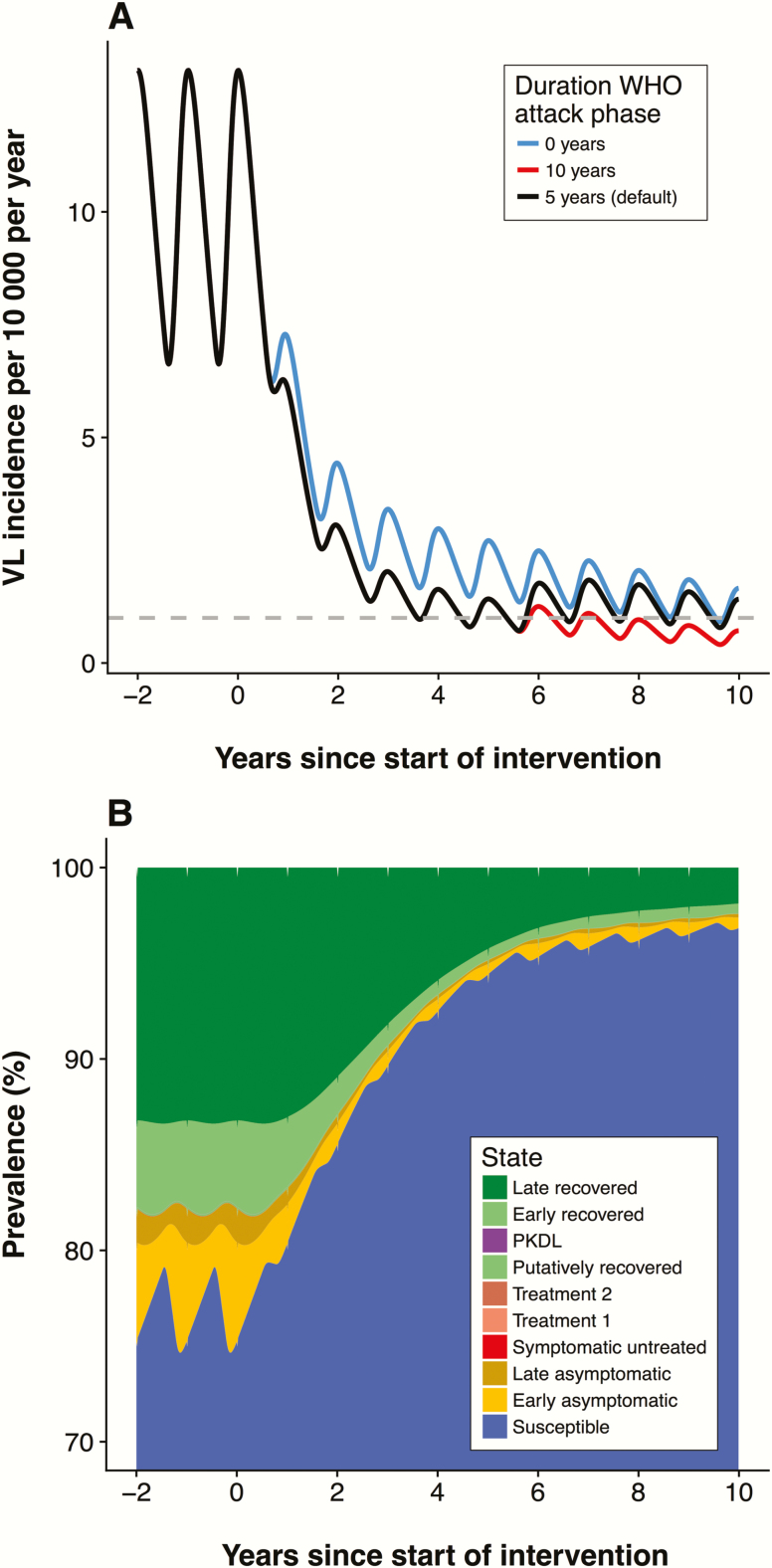
*A,* Predictions from model E0 for the default duration (5 years) and 2 alternative durations (10 years and 0 years) of the attack phase for a setting with a high precontrol endemicity (10 cases per 10000 people per year). Supplementary Figure 1 includes these predictions from all 4 models for 3 different precontrol endemicity settings. *B*, Stacked line chart of the distribution of infection states over time for model E0, in a setting with a high precontrol endemicity (10 cases per 10000 people per year) with the default 5-year attack phase starting in year 0, followed by the consolidation phase. Supplementary Figure 2 includes the distribution of infection states over time for all 4 models in a high-endemicity setting. Abbreviations: PKDL, post-kala-azar dermal leishmaniasis; VL, visceral leishmaniasis; WHO, World Health Organization.

Figure 2 shows that the attack phase brings down the incidence quickly, which is then sustained by the consolidation phase. If the IRS efficacy is relatively low (models W0 and W1), reducing the IRS coverage after 5 years does not appear to have a big impact on incidence, as incidence decreases to very low levels in most settings after 5 years of ACD and intensive IRS. Only model E0 in the highly endemic precontrol setting (10 per 10000 per year) suggests that the target will not be reached within 10 years with the current strategy. We also explored alternative durations of the attack phase for the 3 precontrol endemicity levels, as presented in Figure 3A for model E0 (see Supplementary Figure 1 for outcomes for the other models). Increasing the duration of the attack phase in high precontrol endemicity settings from 5 to 10 years brings forward the elimination target by at least 5 years for this model (E0), but less so for the other models and endemicity levels (see Supplementary Figure 1). Doing this for a medium-endemicity setting gives hardly any additional benefit in the short term and does not result in reaching the target earlier. In contrast, for low-endemicity settings, all models predict that leaving out the attack phase entirely, and starting with the consolidation phase, at worst leads to a minor increase in time to elimination. Adjusting the length of the attack phase to the precontrol endemicity level may therefore lead to more effective use of limited resources.

The distribution of the different disease states of model E0 over time is presented in Figure 3B, which emphasizes the large susceptible population (blue) that accumulates when nearing and sustaining elimination, posing a risk factor for (re-)introduction and recrudescence of infection.

## ROLE OF PKDL IN MAINTAINING TRANSMISSION

**Figure 4.**
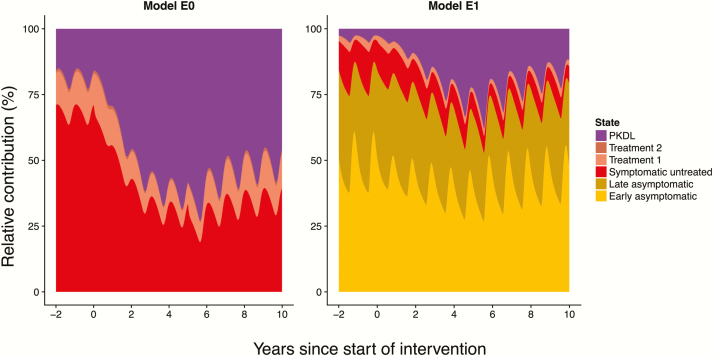
Relative contribution of different disease states to visceral leishmaniasis (VL) transmission over time during the World Health Organization–recommended interventions. In model E0 (left), only symptomatic individuals (VL and post-kala-azar dermal leishmaniasis [PKDL]) contribute to transmission. In model E1 (right), asymptomatic individuals are the main contributors to transmission. Both graphs are for a 10 per 10000 persons per year precontrol endemicity setting with 5-year attack phase followed by the consolidation phase. Assumptions: 2.5% of treated VL cases develop PKDL, PKDL lasts for 5 years on average, and PKDL cases are half as infectious as active VL cases. Supplementary Figure 3 includes the relative contribution of different disease states for all 4 models.

The potential role of PKDL in maintaining transmission as VL incidence decreases is illustrated by Figure 4, which shows the change in the contribution of different disease states to transmission during the WHO phases for models E0 and E1. The relative contribution of PKDL increases as the elimination target is approached. After 5 years of the attack phase, about 70% (model E0) or 25% (model E1) of the infection pressure to sandflies comes from PKDL cases. Both models show that the role of PKDL increases when nearing elimination [4, 13]. Active detection and treatment of PKDL cases would thus be a promising additional tool to speed up and sustain elimination, but diagnosis of PKDL remains a challenge [32, 33].

## DISCUSSION

Our models suggest that the 4-phase intervention strategy, as described in the current WHO guidelines, is likely to be sufficient to reach the target of elimination as a public health problem in areas that had low to medium VL endemicities (≤5 cases per 10000 people per year) prior to the start of interventions. For higher precontrol endemicities, an extended attack phase (intensive IRS and ACD) may be required. Maintaining the target level of incidence might require the same level of interventions that was required to achieve it. Whether IRS or reducing onset-to-treatment time is the more effective intervention depends on the relative infectiousness of asymptomatic and symptomatic individuals and the efficacy of IRS, both still important gaps in knowledge. If most asymptomatic individuals are infectious to sandflies (even if only 1/80th as infectious as symptomatic individuals [19]) and their duration of infection is as long and their rate of developing VL as low as estimated, then they will act as the main source of transmission. In this case, increasing IRS coverage will cause a greater reduction in transmission than reducing delays to treatment, provided IRS is effective in killing sandflies [15]. However, if asymptomatic individuals are not infectious to sandflies, or only a very small proportion of them are, so that clinical cases drive transmission, then reducing delays to treatment will lead to a greater decrease in incidence [28]. These varying assumptions, some of which are covered by the different submodels, influence the time taken to reach elimination.

In the model outputs, the decrease in VL incidence is solely attributable to the impact of interventions. However, the impact of the main interventions on VL incidence, or, in the case of IRS, even on vectorial capacity, varies significantly between studies [34–37]. Reported VL incidence in the Indian subcontinent has declined considerably since 2011, from approximately 37000 cases to just 6500 in 2016 [38]. The decline is likely multifactorial; some have attributed it to improved vector control and others to reductions in delays to treatment [39, 40]. However, other factors that should be borne in mind include a possible natural cycle of VL in the community and the effect of herd immunity [1, 41], alongside a decrease in risk factors for developing the disease, such as malnutrition.

The models reveal the potential hurdle that asymptomatic individuals and, in particular, PKDL cases may pose to reaching and sustaining the target, which is currently not addressed in the elimination target or general strategy. As already suggested by the WHO [10], we recommend including PKDL cases in the VL elimination strategy and target, for which a combined detection strategy, for example together with leprosy, may offer an efficient and sustainable solution. Adding PKDL in the elimination target, however, requires some empirical threshold that has not yet been established [10].

Current models have proven to be useful tools for evaluating broad-scale trends in VL incidence under different assumptions about the underlying biology. As incidence falls, the influence of stochastic effects will increase, so a stochastic individual-based model will be required to predict the probability of true elimination or resurgence in the maintenance phase, and will be included in future studies. When incidence decreases further, different (largely unknown) aspects of the transmission become increasingly important, such as the highly focal nature of the disease, the constant migration of individuals, and potential “super-spreading” of infection by human immunodeficiency virus–coinfected patients [42]. Alternative, sustainable vector control interventions, which are available now, and new tools such as a vaccine against VL or PKDL, which might become available in the future, could help to reduce incidence and sustain the target of elimination as a public health problem. The potential impact of different types of vaccines is currently being explored by the Erasmus team.

## CONCLUSIONS

Modeling analyses suggest that the current WHO strategic guidance seems adequate to reach the target of elimination of VL as a public health problem in areas that had medium precontrol endemicities (up to 5 per 10000 per year) before active case detection and high-coverage IRS began, but that additional interventions may be required in areas with higher precontrol endemicities, such as a longer duration of the attack phase. Asymptomatic individuals and PKDL cases pose a potential threat to reaching and sustaining elimination, leading to ongoing “hidden” transmission for several years after reaching the target. This needs to be addressed in the elimination target and strategy. Also, the increasing pool of susceptible individuals that forms as VL incidence decreases may be a source of new epidemics.

## Supplementary Data

Supplementary materials are available at *Clinical Infectious Diseases* online. Consisting of data provided by the authors to benefit the reader, the posted materials are not copyedited and are the sole responsibility of the authors, so questions or comments should be addressed to the corresponding author.

Supplementary File 1

Supplementary Figure 1

Supplementary Figure 2

Supplementary Figure 3

## Notes

***Acknowledgments.*** The authors gratefully acknowledge the Bihar Technical Support Program of CARE India and the KALANET consortium for providing the data with which the transmission models presented here were originally fitted and validated.

***Financial support.*** This work was supported by the Bill & Melinda Gates Foundation in partnership with the Task Force for Global Health through the Neglected Tropical Disease Modelling Consortium (grant number OPP1053230).

***Supplement sponsorship.*** This article appears as part of the supplement “Reaching the 2020 Goals for Nine Neglected Tropical Diseases,” sponsored by the NTD Modelling Consortium.

***Potential conflicts of interest.*** P. L. O. and J. A. R.-P. are staff members of the WHO. All other authors report no potential conflicts of interest. All authors have submitted the ICMJE Form for Disclosure of Potential Conflicts of Interest. Conflicts that the editors consider relevant to the content of the manuscript have been disclosed.
