# Supplementary material for: Policy Recommendations From Transmission Modeling for the Elimination of Visceral Leishmaniasis in the Indian Subcontinent
Source: Clin Infect Dis. 2018 Jun 1;66(Suppl 4):S301–8. doi: 10.1093/cid/ciy007 (PMC5982727; doi:10.1093/cid/ciy007)
Supplement: Supplementary File 1 [file ciy007_suppl_supplementary_file_1.pdf]

Supplementary File 1: Links to model details, code and uncertainty and sensitivity analyses

1. Details of the models

For full details of the Erasmus MC models (models E0 and E1) see Additional File 1 of [1] and [2], and for a full description of model W1 see Supplementary File S1 of [2]. Model W0, introduced in this study, is the same as model W1 except that it assumes asymptomatic individuals are not infectious. Details of validation and comparison of all four models are available in [2].

2. Model code

The code for the Erasmus MC models (E0 and E1) used to produce the results in the paper is available at http://ars.els-cdn.com/content/image/1-s2.0-S1755436516300792-mmc5.zip, that for the Warwick models (W0 and W1) is available at http://wrap.warwick.ac.uk/95590/.

3. Parameter uncertainty and sensitivity analyses

Detailed parameter sensitivity and uncertainty analyses for models E0, E1, and W1 can be found in [1,2] and their associated supplementary material.

References

1. Le Rutte EA, Coffeng LE, Bontje DM, et al. Feasibility of eliminating visceral leishmaniasis from the Indian subcontinent: explorations with a set of deterministic age-structured transmission models. Parasit. Vectors 2016; :1–14. Available at: http://dx.doi.org/10.1186/s13071-016-1292-0.

2. Le Rutte EA, Chapman LAC, Coffeng LE, et al. Elimination of visceral leishmaniasis in the Indian subcontinent: a comparison of predictions from three transmission models. Epidemics 2017; 18:67–80. Available at: http://dx.doi.org/10.1016/j.epidem.2017.01.002.
