# Supplementary material for: Policy Recommendations From Transmission Modeling for the Elimination of Visceral Leishmaniasis in the Indian Subcontinent
Source: Clin Infect Dis. 2018 Jun 1;66(Suppl 4):S301–8. doi: 10.1093/cid/ciy007 (PMC5982727; doi:10.1093/cid/ciy007)
Supplement: Supplementary Figure 1 [file ciy007_suppl_supplementary_figure_1.pdf]

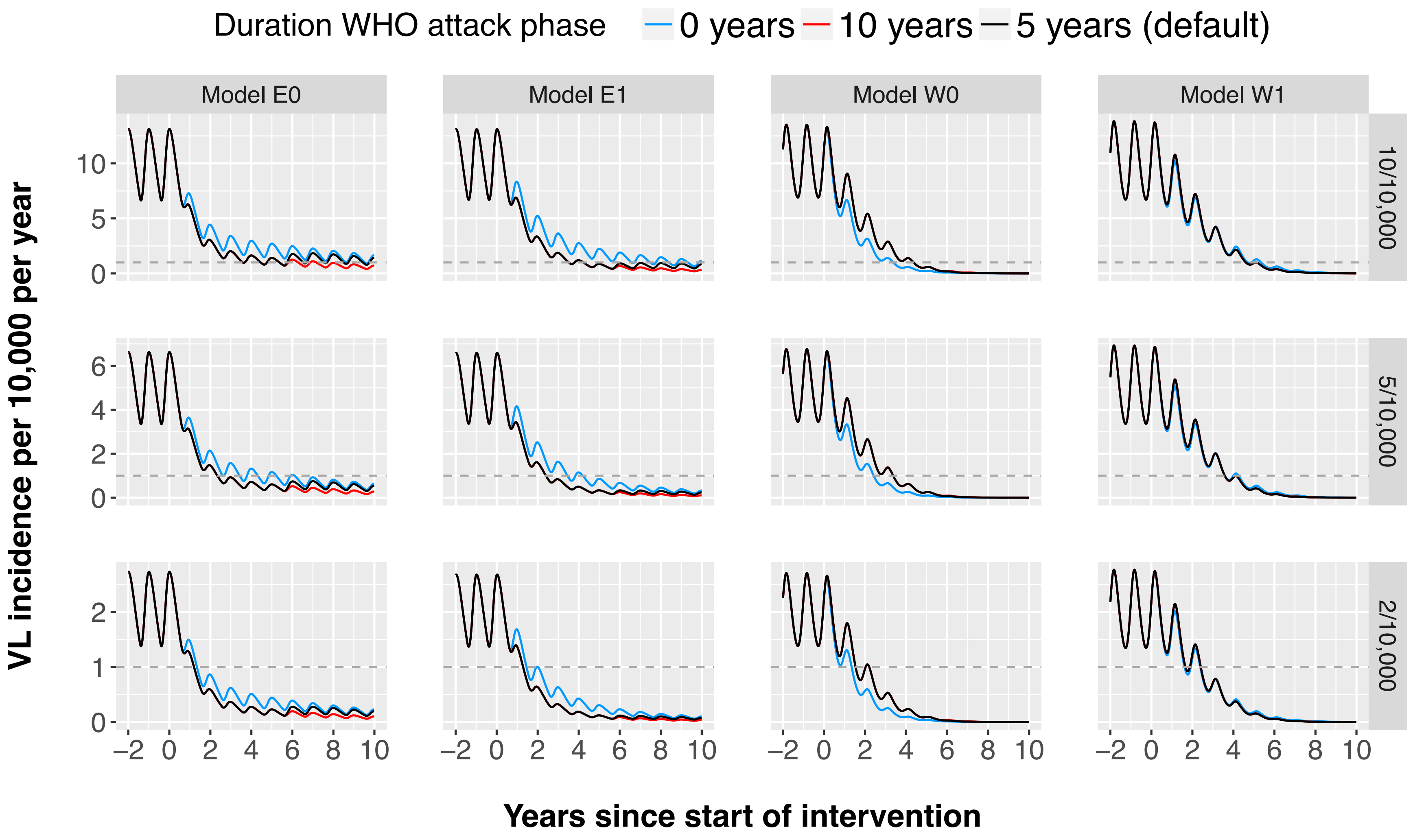

Supplementary Figure 1. Predictions of VL incidence from all four models for different durations of the WHO attack phase (0, 5 and 10 years) for three different pre-control endemicities (2, 5 and 10 cases/10,000 people/yr). The grey dashed line represents the 1/10,000/yr elimination target. The predicted impact of changing the duration of the attack phase is different for the different models. For models E0 and E1, extending the attack phase from 5 to 10 years leads to a faster reduction in incidence, and removing the attack phase to a slower reduction in incidence, at all endemicity levels. In contrast, for models W0 and W1, doubling the attack phase duration to 10 years has very little impact on the rate at which incidence decreases for all pre-control endemicity levels, and perhaps counter-intuitively removing the attack phase leads to a faster reduction in incidence for model W0 and only a very slightly slower reduction in model W1. Models E0 and E1 also show a small ‘bounce back’ in incidence when the assumed IRS coverage is reduced after the attack phase, whereas there is little observable bounce back for models W0 and W1. These differences are due to differences between the models in the asymptomatic contribution to transmission, the much lower estimated IRS efficacy used in the Warwick models than the Erasmus models (such that reducing the onset-to-treatment time is predicted to have a bigger impact than increasing IRS coverage), and the inclusion of PKDL in the Erasmus models.
