# Supplementary material for: Policy Recommendations From Transmission Modeling for the Elimination of Visceral Leishmaniasis in the Indian Subcontinent
Source: Clin Infect Dis. 2018 Jun 1;66(Suppl 4):S301–8. doi: 10.1093/cid/ciy007 (PMC5982727; doi:10.1093/cid/ciy007)
Supplement: Supplementary Figure 2 [file ciy007_suppl_supplementary_figure_2.pdf]

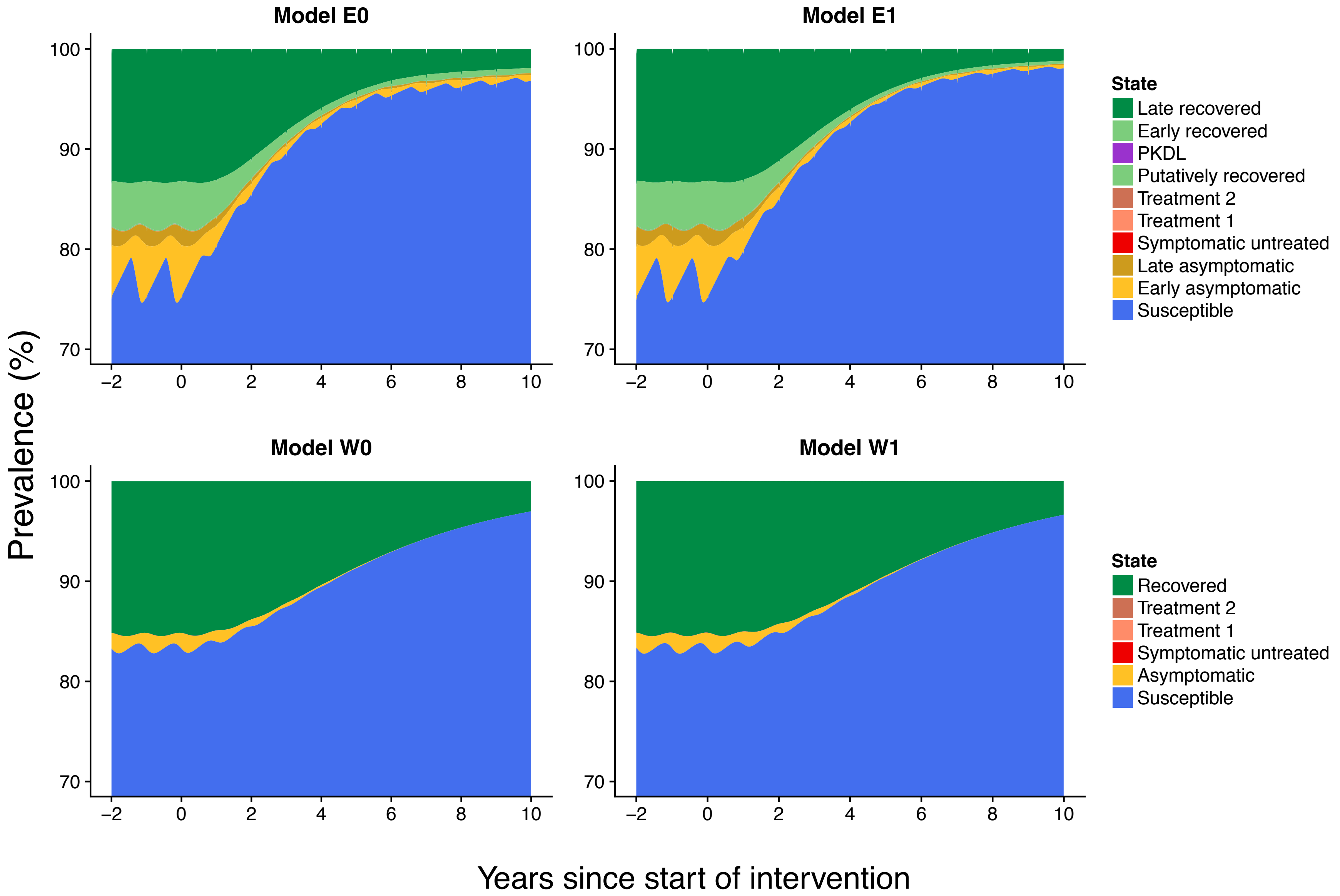

Supplementary Figure 2. Stacked line charts of the distribution of infection states over time for all four models, in a setting with a high pre-control endemicity (10 cases/10,000 people/yr) with the default 5-year attack phase starting in year 0, followed by the consolidation phase.
