# Supplementary material for: Policy Recommendations From Transmission Modeling for the Elimination of Visceral Leishmaniasis in the Indian Subcontinent
Source: Clin Infect Dis. 2018 Jun 1;66(Suppl 4):S301–8. doi: 10.1093/cid/ciy007 (PMC5982727; doi:10.1093/cid/ciy007)
Supplement: Supplementary Figure 3 [file ciy007_suppl_supplementary_figure_3.pdf]

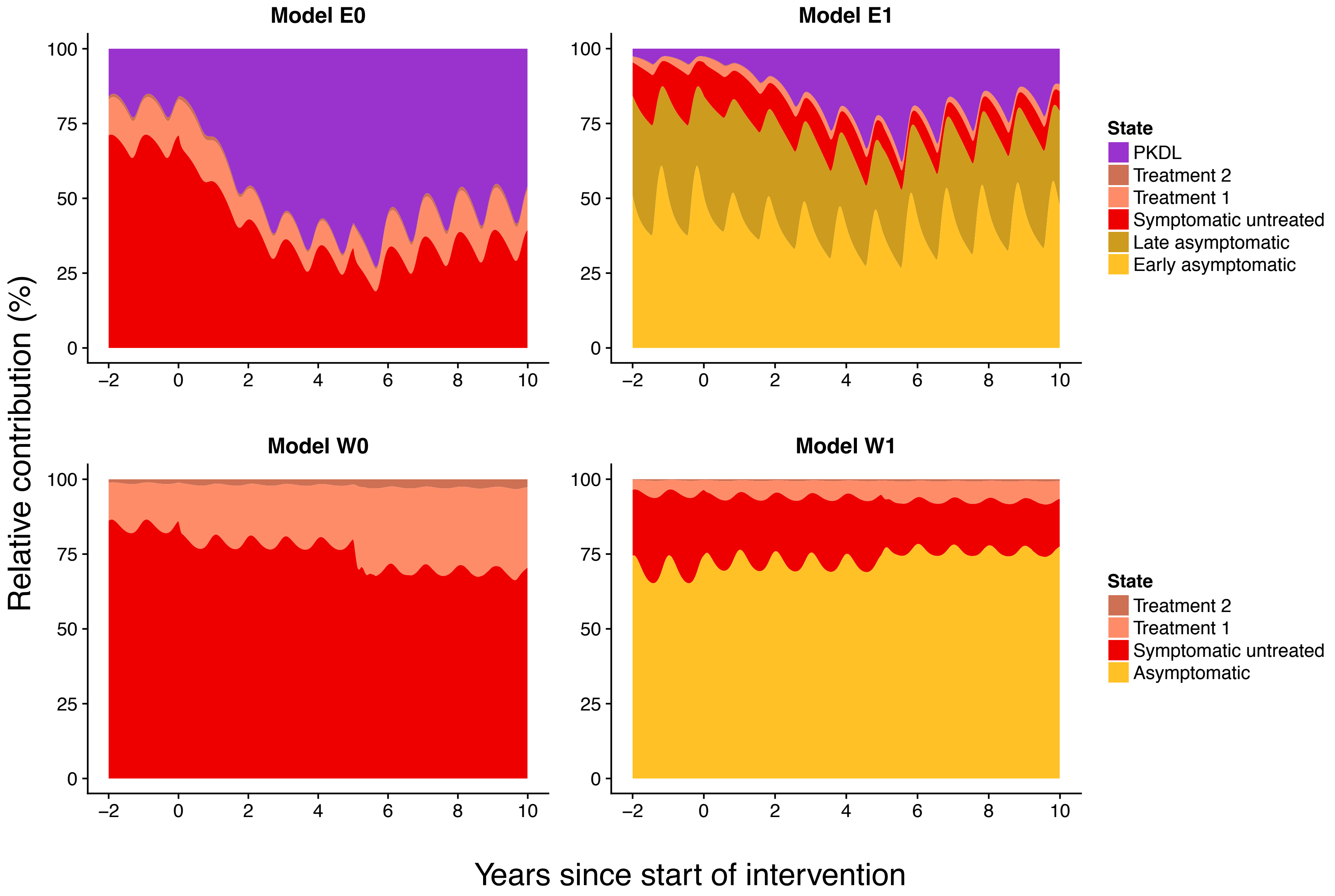

Supplementary Figure 3. Relative contribution of different disease states to VL transmission over time during the WHO-recommended interventions for all four models. In models E0 and W0 (left), only symptomatic individuals contribute to transmission. In models E1 and W1 (right), asymptomatic individuals are the main contributors to transmission. All graphs are for a 10/10,000/yr pre-control endemicity setting with a 5-year attack phase followed by the consolidation phase.
